# Sands of Sahara after LASIK in Avellino Corneal Dystrophy

**DOI:** 10.1155/2012/413010

**Published:** 2012-02-02

**Authors:** Flavio Mantelli, Alessandro Lambiase, Antonio Di Zazzo, Stefano Bonini

**Affiliations:** Department of Ophthalmology, Campus Bio-Medico University of Rome, Via Alvaro del Portillo 21, 00128 Rome, Italy

## Abstract

We report the case of a patient diagnosed with Avellino corneal dystrophy (ACD) who developed diffuse interstitial keratitis following excimer laser insitu keratomileusis (LASIK). ACD is an autosomal dominant corneal dystrophy characterized by multiple asymmetric stromal opacities that impair vision. Accepted treatments for this condition include corneal transplantation and phototherapeutic keratectomy (PTK). Our patient underwent LASIK at another institution to correct myopia. LASIK and photorefractive keratectomy (PRK) are usually contraindicated in ACD for the high risk of disease recurrence and postoperative complications. The patient came to our attention lamenting blurry vision, decreased visual acuity, and photophobia. Ophthalmologic examination revealed bilateral interstitial keratitis, also known as “sands of Sahara”, a seldom-seen complication of LASIK characterized by fine and diffuse granular infiltrates at the surgical flap interface.The risk of developing interstitial keratitis, as in the case presented here, represents another valid reason for avoiding LASIK in patients with ACD.

## 1. Introduction

Avellino corneal dystrophy (ACD), also known as Granular Corneal Dystrophy type2, has been first described in three families from Avellino, Italy, and it has since been reported all around the world [1]. It is an autosomal dominant corneal disease characterized by asymmetric grey-white central anterior stromal opacities of various shape and size and deep lattice-like stromal deposits [2]. Early clinical symptoms of ACD appear during the first or second decade of life; however, compared to Granular Corneal Dystrophy type I, the progression of ACD is delayed and slowed, and the visual acuity is less impaired [3]. Therefore, surgical therapeutic intervention for this corneal dystrophy is largely debated, with several arguments for and against. Currently accepted treatments for ACD include excimer laser phototherapeutic keratectomy (PTK) and corneal transplantation [4]. PRK and LASIK are contraindicated for the usual recurrence of disease and for the additional risk of postoperative complications impairing corneal transparency [5, 6]. Specifically, several reports highlighted how LASIK treatment induces moderate-to-severe early exacerbations of the disease and a marked acceleration in disease course with rapidly worsening visual outcome [6].

In this case report, presented with the consent of the patient in accordance with the tenants of the Declaration of Helsinki, we describe a seldom-seen complication of LASIK performed in a patient previously diagnosed with ACD.

## 2. Case Presentation

An otherwise healthy 45-year-old Caucasian woman, with a previous diagnosis of ACD, presented to our cornea and external eye disease unit lamenting a progressive decrease in vision in both eyes in the last year accompanied by photophobia and difficulty in driving at night. The patient reported that her visual acuity has been worsening since she performed bilateral laser vision correction (LASIK) approximately 15 months earlier at another institution to correct myopia. No additional data was provided on the modalities of laser ablation. At the time of surgery, the patient was informed that the laser vision correction would have also eliminated the stromal opacities caused by ACD.

**Figure 1:**
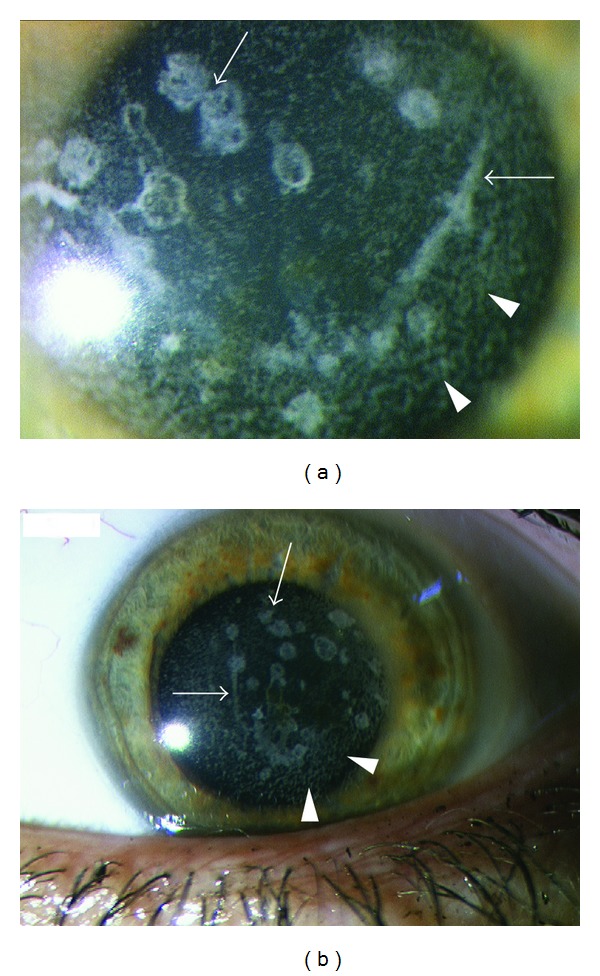
Slit-lamp examination shows multiple bilateral (a: right eye; b: left eye) crumb-like and lattice-like opacities of the stroma (arrows) and multiple foci of fine granular infiltrates at the surgical flap interface (arrowheads).

**Figure 2:**
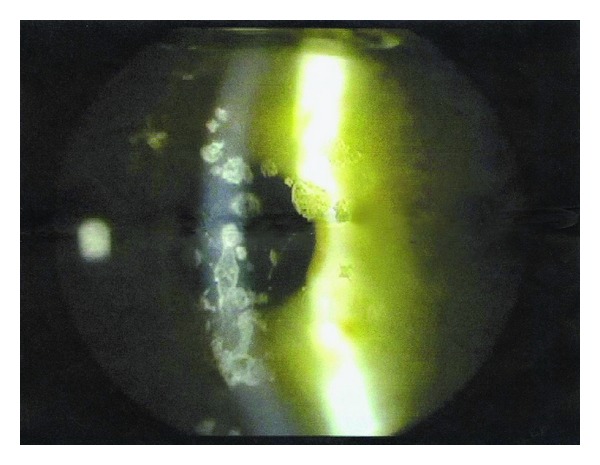
A digitalized picture of the right eye, taken before LASIK was performed, shows the presence of multiple crumb-like and lattice-like stromal opacities consistent with the diagnosis of ACD. No granular infiltrates were present before surgery.

Best corrected visual acuity of our patient before surgery reportedly was 20/25 in the right eye and 20/20 in her left eye. Ophthalmologic examination revealed a corrected visual acuity of 20/40 in the right eye and 20/32 in the left eye. Slit-lamp examination (Figure 1(a): right eye; 1(b): left eye) revealed multiple crumb-like and lattice-like opacities of the corneal stroma (arrows), consistent with the diagnosis of Avellino dystrophy. As compared to the pre-LASIK image of the right eye kindly provided by the patient (Figure 2), multiple foci of fine granular infiltrates at the surgical flap interface (arrowheads, Figure 1) were also observed in our patient.

Dilated fundus oculi examination was normal. All medical treatments prescribed after the LASIK procedure, including topical corticosteroids, were unsuccessful in improving vision. The patient is currently waiting bilateral corneal transplant.

## 3. Discussion

LASIK has become the refractive procedure of choice for myopia due to its quick recovery time and low rate of complications; however, its indications in patients affected by corneal dystrophies are still largely debated. In fact, during LASIK the microkeratome pass through the stromal layers results in an interface in which severed collagen fibers lie in apposition without any barrier [7]. This interface might facilitate the deposition of abnormal proteins to the damaged collagen fibers, resulting in a rapid appearance of corneal infiltrates. These corneal infiltrates are a seldom-seen postoperative complication of LASIK known as “diffuse interstitial keratitis” or “Sands of Sahara”, causing blurred vision, sensitivity to bright ambient lighting, and permanent reduction in visual acuity [8]. They reflect a noninfectious diffuse flap interface inflammatory response of the corneal stroma happening after lamellar corneal surgery [9]. The increased risk of early recurrence of the granular stromal deposit after LASIK represents a well-known complication in patients with ACD [10]. The additional risk of developing a diffuse interstitial keratitis, as shown in the case reported here, represents another valid reason for avoiding LASIK in patients with ACD and, generally, in patients with stromal corneal dystrophies, to avoid complex postoperative managements and painful legal consequences.
